# Early EEG and serum biomarkers for prognostication after cardiac arrest

**DOI:** 10.1016/j.resplu.2025.101154

**Published:** 2025-11-06

**Authors:** Inken Alina Strate, Johannes G. Krabbe, Sjoukje Nutma, Albertus Beishuizen, Wytze J. Vermeijden, Francois H.M. Kornips, Norbert A. Foudraine, Jeannette Hofmeijer, Michel J.A.M. van Putten

**Affiliations:** a Clinical Neurophysiology (CNPH), Technical Medical Center, University of Twente, Drienerlolaan 5, 7500 AE Enschede, the Netherlands; b Department of Clinical Chemistry and Laboratory Medicine, Medlon BV, Enschede, the Netherlands; c Department of Clinical Chemistry and Laboratory Medicine, Medisch Spectrum Twente, Enschede, the Netherlands; d Department of Clinical Neurophysiology and Neurology, Medisch Spectrum Twente, Koningsplein 1, 7512 KZ Enschede, the Netherlands; e Department of Intensive Care Medicine, Medisch Spectrum Twente, Enschede, the Netherlands; f Department of Neurology, VieCuri Medical Center, P.O. Box 1926, 5900 BX Venlo, the Netherlands; g Department of Critical Care, VieCuri Medical Center, Venlo, the Netherlands; h Department of Neurology, Rijnstate Hospital, Wagnerlaan 55, 6815 AD Arnhem, the Netherlands

**Keywords:** Biomarkers, Prognostication, Neurological outcome, Cardiac arrest, EEG

## Abstract

**Rationale:**

Early electroencephalography (EEG), initiated within 12 h after cardiac arrest, allows reliable prognostication in approximately 50 % of comatose patients. Serum biomarkers may complement early EEG–based outcome prediction, particularly in indeterminate cases. We evaluated the potential additive prognostic value of the serum biomarkers neuron-specific enolase (NSE), neurofilament light chain (NfL), S100 calcium binding protein *β* (S100B), and phosphorylated tau (p-Tau 181) when combined with early EEG.

**Methods:**

In this pilot study, we analysed serum biomarker concentrations at multiple time points (<12 h, 24 h, 72 h, 7 days) post-CA in comatose out-of-hospital CA patients included in the ghrelin in coma (GRECO) trial. EEG recordings were visually evaluated at 12 and 24 h post-arrest. Neurological outcomes were assessed using the Cerebral Performance Category (CPC) score at 6 months, dichotomised into good (CPC 1–2) or poor (CPC 3–5).

**Results:**

A total of 49 patients were included; 24 (49 %) had a poor neurological outcome at 6 months. Serum biomarker concentrations were significantly higher in poor-outcome patients within 24 h post-CA. NfL achieved an AUC of 0.90, followed by p-Tau181 (AUC 0.81), S100B (AUC 0.80) and NSE (AUC 0.76) for poor neurological outcome at 24 h post-CA. In 40 patients with EEG, NfL > 100 pg/mL reclassified indeterminate cases as likely poor outcome, with >128 pg/mL at any time achieving 100 % specificity, unlike other biomarkers.

**Significance:**

NfL demonstrated superior prognostic performance compared to other serum biomarkers. Our preliminary findings suggest added prognostic value of NfL when combined with early EEG (12–24 h), particularly in patients with indeterminate EEG findings.

## Introduction

Cardiac arrest remains a major cause of death and disability and up to half of survivors admitted to the ICU experience a poor neurological outcome.^1^ Accurate early prognostication is critical to guide treatment decisions and family counselling, and current guidelines recommend a multimodal approach including neurological examination, EEG, somatosensory evoked potentials, imaging, and serum biomarkers.^2^

Early EEG, obtained within 24 h, allows reliable prediction in about half of patients at very high specificity, but leaves a substantial proportion in a prognostic “grey zone” with indeterminate patterns.^3^, ^4^, ^5^, ^6^, ^7^, ^8^, ^9^ Serum biomarkers may complement EEG by reflecting the extent of brain injury.^10^, ^11^, ^12^ biomarkers such as NSE and S100 calcium-binding protein beta (S100B) have been extensively investigated across various brain injuries,^13^, ^14^ newer biomarkers like neurofilament light chain (NfL) and phosphorylated tau (p-Tau181) are increasingly recognised for their potential prognostic properties.^2^, ^15^, ^16^, ^12^ These biomarkers reflect distinct aspects of neurovascular unit injury: neuronal cell bodies (NSE), axons (NfL, tau), and glia (S100B).^10^, ^12^ Neurofilament light chain (NfL) has emerged as the most promising marker of axonal damage, with strong performance across multiple cohorts.^17^, ^12^, ^18^, ^19^, ^20^

Associations between serum biomarkers and early EEG (initiated within 12 h), including NfL, and their potential additive prognostic value, have not yet been reported. Against this background, we aimed to evaluate the prognostic contribution of four brain biomarkers (NSE, NfL, S100B, p-Tau181) in relation to standardised EEG within 24 h after cardiac arrest, with a specific focus on whether biomarkers can improve outcome prediction in patients with indeterminate EEGs.

## Methods

### Study design and population

In this retrospective analysis of prospectively collected data, we analysed patient data and serum specimens of adult comatose patients (GCS < 7 at ICU admission) after cardiac arrest from the Ghrelin in Coma (GRECO) prospective clinical trial, registered at clinicaltrialsregister.eu (EUCTR2018-000005-23-NL), conducted between 2019 and 2022 across three intensive care units (Medisch Spectrum Twente, Universitair Medisch Centrum Groningen, VieCurie Medical Centre Venlo) in the Netherlands. Patients with known progressive neurological disease and expected death within 48 h (based on clinical judgment of irreversible organ failure) after admission were excluded. Detailed inclusion and exclusion criteria for the GRECO trial are described elsewhere.^21^ Standard clinical care was provided in accordance with Dutch and European guidelines. Withdrawal of life-sustaining treatment was considered if patients remained comatose after cessation of sedation (Glasgow Coma Scale (GCS) score <7) and met one or more of the following criteria: burst suppression with identical bursts on the EEG at any time point, a suppressed EEG background at 24 h after cardiac arrest, bilaterally absent SSEP or absent pupillary reflexes ≥24 h. Serum biomarker findings were not available to the treating physicians.

### Biomarker measurements

Serum samples were collected at ≤12 h, 24 h, 72 h, and 7 days after arrest and stored at −80 °C. Concentrations of NSE, NfL, S100B, and p-Tau181 were measured using validated immunoassays under standard laboratory procedures, subjected to a single freeze–thaw cycle, and measured in singlicate. NSE and S100B concentrations were determined using the Cobas® e801 module of the Cobas® 8000 analyzer (Roche Diagnostics GmbH, Mannheim, Germany). Samples with a haemolysis index >50 were excluded from NSE analysis. p-Tau181 and NfL concentrations were measured using the LUMIPULSE® G1200 system (Fujirebio Holdings Inc., Tokyo, Japan). Precision verification of all biomarkers was conducted in accordance with CLSI EP5-A3 or CLSI EP15-A2 guidelines. The total coefficient of variation (CV) for all biomarkers was below 8 %. Additionally, internal controls and proficiency testing ensured consistency throughout the sample measurements.

### EEG analysis

Continuous EEG was started within 12 h and reviewed at 12 h and 24 h by two blinded raters using ACNS criteria.^22^ Patterns were categorised into three EEG groups: (I) unfavourable patterns (*≥*24 h suppressed (<5 µV), time-independent burst-suppression with identical bursts and generalised periodic discharges on a suppressed background); (II) indeterminate/uncertain patterns (low-voltage (5–20 uV), discontinuous, evolving electrographic seizures, burst-suppression without identical bursts, generalised periodic discharges on other backgrounds); and (III) favourable EEG patterns (continuous activity diffusely slowed <8 Hz, or normal >8 Hz) at 12 h and 24 h post-cardiac arrest. Discrepancies between raters were resolved by consensus. Neurological outcome at 6 months was assessed with the Cerebral Performance Category (CPC), dichotomised into good (CPC 1–2) or poor (CPC 3–5).

### Outcome assessment

Neurological outcome was assessed at 6 months using the Cerebral Performance Category (CPC) scores, with CPC 1–2 defined as a good outcome (from full recovery to moderate disability) and CPC 3–5 as a poor outcome (from severe disability to death). Assessment of neurological outcome was performed blinded to EEG classes and biomarker results.

### Statistical analysis

Group comparisons used non-parametric statistics, and ROC curves quantified prognostic performance. Comparisons between biomarker levels were adjusted for multiple testing using Bonferroni correction. A *p* < 0.05 was considered significant. Optimal cut-off values were determined at 100 % specificity for poor outcome. All analyses were performed using GraphPad Prism for MacOS (version 10.0, GraphPad Software, San Diego, CA).

## Results

**Fig. 1:**
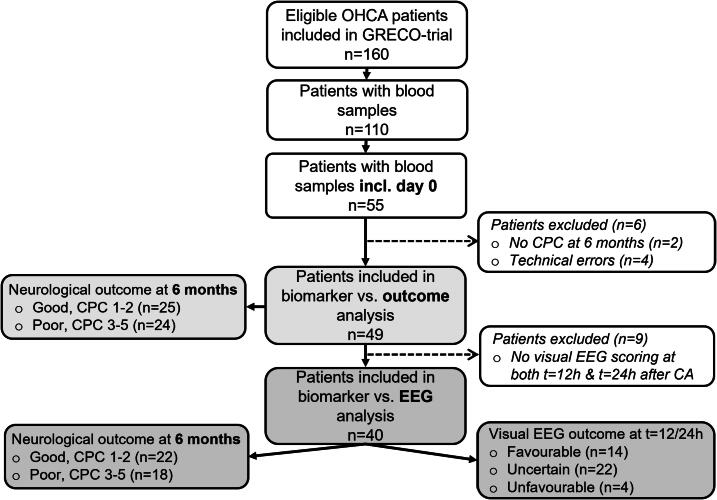
Flowchart of patient inclusion. Day 0 is defined as ≤12 h post-cardiac arrest (CA). CPC, Cerebral Performance Category; EEG, electroencephalography; OHCA, out-of-hospital cardiac arrest.

Of 160 patients in the GRECO trial, 49 had biomarker samples and outcome data available; 40 also had EEG recordings. Of the 40 patients with EEG, 22 (55 %) were classified as having a good neurological outcome (13 = CPC 1, 9 CPC = 2), while 18 (45 %) had a poor neurological outcome (3 CPC = 3, 0 CPC = 4, 15 CPC = 5) at 6 months post-arrest. See Fig. 1 for patient distribution.

Most patients with a CPC = 5 died from WLST based on a poor neurological outcome. Three patients died from non-neurological causes. The median age of patients with a good outcome was significantly lower (66 years) than those with a poor outcome (77 years, *p* = 0.008). At 6 months, 22 patients had a good outcome and 18 had a poor outcome. Patients with a poor outcome were older, while other baseline characteristics were similar, summarised in Table 1. Ghrelin or placebo, as part of the GRECO trial,^21^ was evenly distributed between outcome groups. No treatment-related differences were observed.

**Table 1:** Baseline characteristics of the analysis subset including 40 patients.

**Outcome**	**Good (CPC 1–2)**	**Poor (CPC 3–5)**
Number	22	18
Median age, years (range)	66 (43–87)	77 (55–88)
Male sex, *n* (%)	17 (77)	8 (44)

**Characteristics of cardiac arrest**		
Cause of cardiac arrest, *n* (%)		
*Cardiac*	21 (95)	16 (89)
*Other*	0 (0)	2 (11)
*Unknown*	1 (5)	0 (0)
Time to ROSC, min (median and IQR)	14 (9–19)	25 (17–34)
WLST, *n* (%)	1 (5)	12 (67)
Treatment with ghrelin, *n* (%)	13 (59)	9 (50)

**EEG**		
Patients EEG pattern ≤24 h, *n* (%)		
*Favourable*	13 (59)	1 (6)
*Indeterminate/Uncertain*	9 (41)	13 (72)
*Unfavourable*	0 (0)	4 (22)

**Biomarkers**		
Taken at time from CA; median and IQR		
Day 0 (*≤*12 h)	6.1 (3.5–9.3)	7.3 (4.5–10.5)
Day 1 (24 h)	23.4 (20.2–31.3)	22.5 (18.2–32.5)

CA, cardiac arrest; CPC, Cerebral Performance Category; h, hours; IQR, interquartile range; ROSC, return of spontaneous circulation; SD, standard deviation; EEG, Electroencephalography; WLST, Withdrawal of Life Sustaining Treatment.

### Biomarker concentrations and neurological outcome

**Fig. 2:**
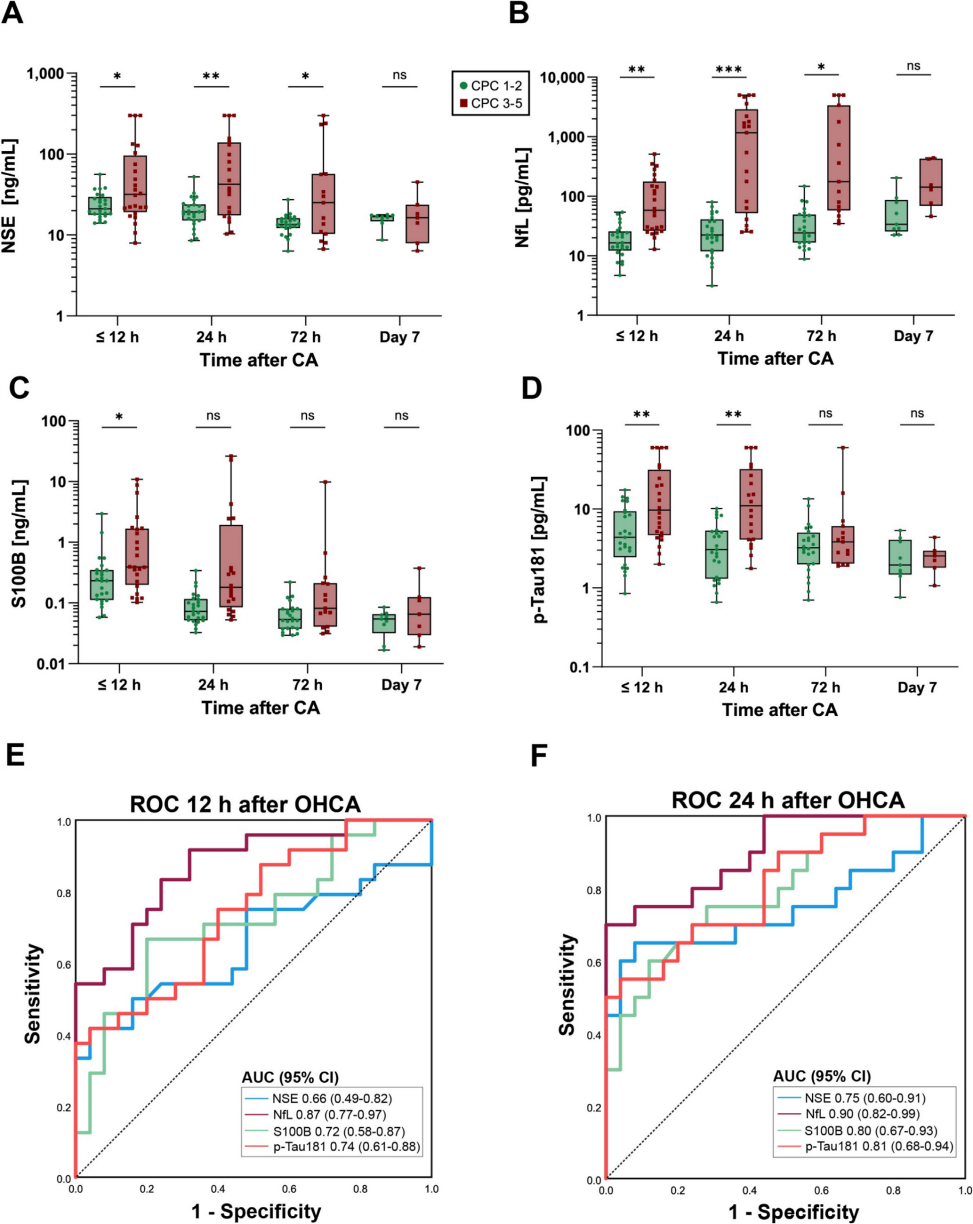
Serum biomarker concentrations between neurological outcome groups across multiple time points. (A–D) Box-and-whisker plots showing serum concentrations stratified by 6-month neurological outcome. Biomarker levels were significantly elevated in patients with poor neurological outcomes across multiple time points, with the most pronounced differences observed within 24 h. (E–F) Receiver operating characteristic (ROC) curves for predicting poor outcome. Number of patient samples per time point: ≤12 h, *n* = 49; 24 h, *n* = 45; 72 h, *n* = 39; Day 7, *n* = 16. ns, not significant; *: *p* < 0*.*05, **: *p* < 0*.*01, ***: *p* < 0*.*001, Mann-Whitney *U* test with Bonferroni correction.

As shown in Fig. 2, all biomarkers were higher in poor outcome patients within the first 24 h, with the clearest separation for NfL. NfL levels in poor outcome patients reached up to 50-fold higher values than in good outcome patients. At 24 h, NfL achieved an AUC of 0.90 (95 % CI 0.82–0.99), outperforming p-Tau181 (0.81), S100B (0.80), and NSE (0.76). A threshold of 128 pg/mL yielded 70 % sensitivity at 100 % specificity for all time-points. If restricted to <24 h, the threshold for a poor outcome at 100 % specificity was 100 pg/ml.

### Combining biomarkers and EEG patterns

**Fig. 3:**
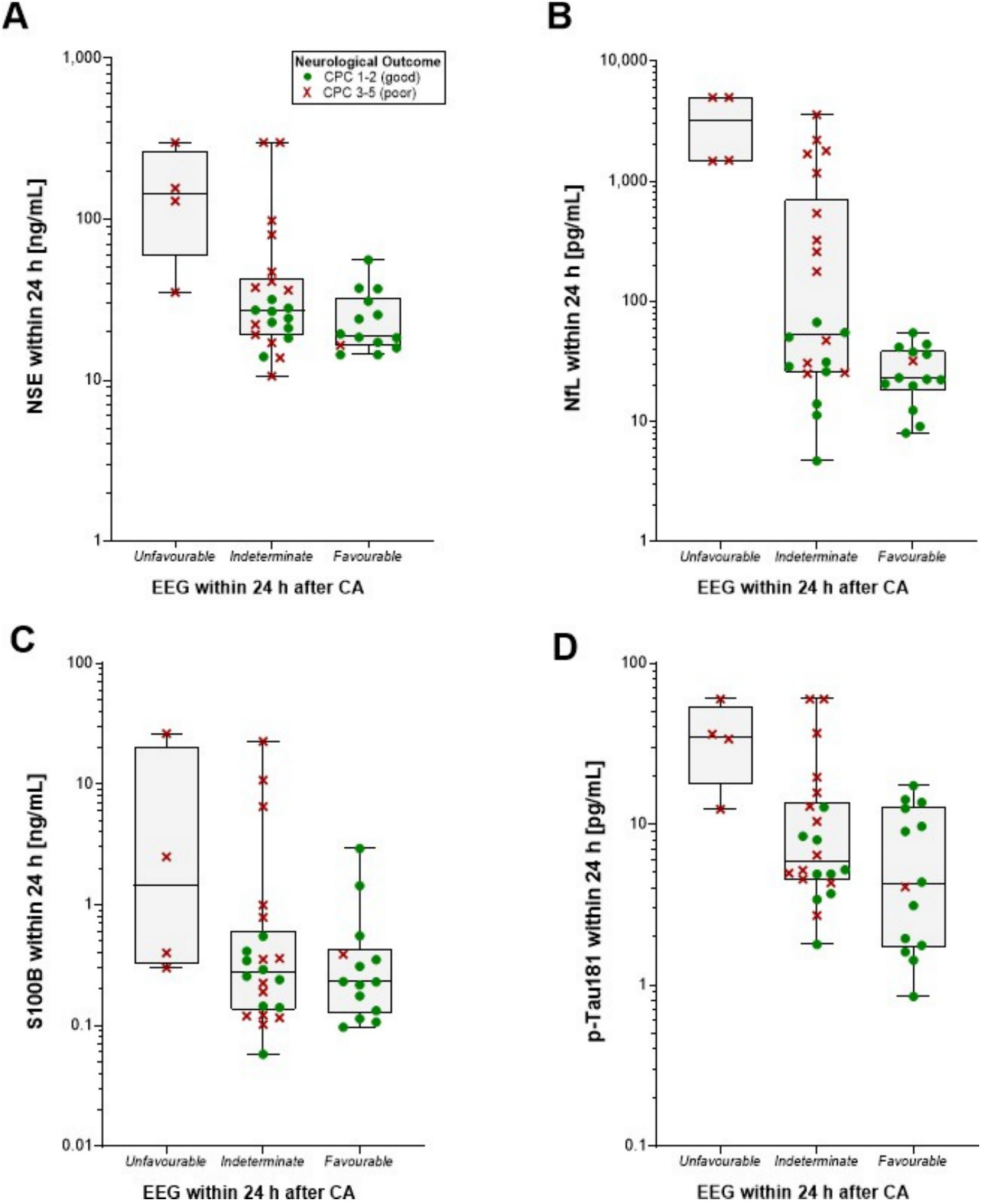
Peak biomarker concentrations present at unfavourable, indeterminate and favourable EEG patterns recorded within 24 h after cardiac arrest and the overall neurological outcome at 6 months in 40 cardiac arrest patients. Dots represent patients with good and crosses those with poor neurological outcomes. In the group with indeterminate EEG, all patients with a NfL *>* 100 pg/mL within 24 h had a poor neurological outcome. The one patient with low biomarker concentrations and a favourable EEG died from a non-neurological cause.

When combined with early EEG (recorded within 24 h post-cardiac arrest), serum biomarkers showed limited additional prognostic value in patients with clearly favourable or unfavourable EEG patterns, as these aligned well with neurological outcomes (CPC 1–2 or 3–5 at 6 months). NfL levels above 1000 pg/mL were observed only in patients with an early EEG indicating poor outcome, whereas NfL below 20 pg/mL occurred exclusively in those with favourable EEGs (Fig. 3B). In patients with indeterminate EEG patterns (*n* = 22), NfL > 100 pg/mL within 24 h identified poor outcome (CPC 3–5) with 100 % specificity and 41 % sensitivity (9/22). Fig. 3 shows extensive overlap for NSE, S100B, and p-Tau181 between outcome groups within each EEG category, in contrast to NfL. These biomarkers did not enhance prognostic accuracy more than NfL in this subgroup (Fig. 3A, C, D). Sensitivities at 100 % specificities in the indeterminate group were 18 % (4/22) for NSE *>* 80 ng/mL, 14 % (3/22) with S 100B *>* 5 ng/mL, and 14 % (3/22) for p-Tau181 *>* 20 pg/mL.

## Discussion

In this pilot study, we evaluated the added value of four serum biomarkers to early (12–24 h) EEGs for prognostication of comatose patients after cardiac arrest. Neurofilament light chain (NfL) consistently outperformed NSE, S100B and p-Tau181, with its incremental value most evident in patients with indeterminate early EEGs. In this subgroup, NfL > 100 pg/mL within 24 h reclassified 41 % of patients towards poor outcome, thereby reducing prognostic uncertainty.

NSE, although guideline-endorsed,^2^ showed wide overlap and limited added prognostic value, consistent with concerns about extracerebral release. S100B and p-Tau181 also performed less well, presumably reflecting their transient or non-specific release dynamics. By contrast, NfL rose early and remained elevated, in line with ongoing axonal injury and previous studies of its kinetics. These findings align with prior reports showing that NfL is a robust serum biomarker for the severity of postanoxic encephalopathy^23^, ^24^, ^25^, ^26^, ^27^, ^20^ and generally achieves higher overall AUCs than EEG when evaluated in isolation. Importantly, our study focused on early EEG (<12 h), which provides functional information at a time when biomarkers are only beginning to rise. The two modalities, therefore, capture different but complementary aspects of brain injury: EEG reflects real-time cortical function, whereas NfL quantifies structural axonal damage. Their integration can thus reduce prognostic uncertainty, particularly in patients with in determinate early EEG patterns.

The motivation for studying the potential added prognostic value of serum biomarkers to EEG recordings started within 12 h after arrest stems from evidence that early EEG carries the most reliable prognostic information, both for poor and good outcomes.^3^, ^7^, ^28^ This complements studies reporting biomarker-EEG associations at later timepoints, such as,^29^ who correlated NSE with EEG at a median of 19 h and,^30^ who linked NfL to EEG at ≥36 h. Early EEG’s high specificity^3^, ^7^ makes it particularly suited for timely assessment, which our biomarker integration aims to enhance. In Dutch practise, EEGs are routinely performed within 12–24 h, whereas final prognostic evaluation occurs only after sedation withdrawal (typically 36–48 h). These early measures may thus inform subsequent multimodal prognostic assessment without implying premature treatment limitation.

The distinct temporal profiles of the biomarkers presumably reflect differences in their cellular origin and release kinetics. Early elevations of p-Tau 181 likely arise from acute cytoskeletal disruption in severely injured neurons, whereas the delayed increases in NSE, S100B, and NfL may result from progressive leakage from glial and axonal compartments as secondary injury evolves. A similar pattern was previously described in 31, supporting our interpretation.

Our study has several limitations. The modest sample size, retrospective biomarker analysis, and imperfect sampling time points limit generalizability. As a post-hoc substudy of the GRECO trial,^21^ the sample was convenience-based and hypothesis-generating, precluding a formal power calculation or separate training/validation cohorts. Unfortunately, biomarkers were only available in a subgroup due to logistical constraints in the GRECO trial. We did not define a predefined threshold for any of the biomarkers to differentiate between poor or good outcome. Ideally, a separate training and validation cohort should be used, but the amount of data available was not sufficient to perform this in a meaningful manner. Although WLST followed strict guidelines and serum biomarker results were unavailable to clinical teams to minimise self-fulfilling prophecy bias, complete exclusion cannot be guaranteed.

In conclusion, our study confirms that NfL is a reliable serum biomarker. It provides preliminary evidence that its greatest value may emerge when combined with early EEG, particularly in patients with indeterminate patterns. These findings support integrating serum biomarkers with early neurophysiology in multimodal prognostication, warranting validation in larger multicentre cohorts.

## Funding source

This research did not receive any specific grant from funding agencies in the public, commercial, or not-for-profit sectors.

## CRediT authorship contribution statement

**Inken Alina Strate:** Writing – original draft, Investigation, Formal analysis, Data curation, Conceptualization. **Johannes G. Krabbe:** Writing – review & editing, Validation, Resources, Data curation. **Sjoukje Nutma:** Writing – original draft, Investigation, Data curation. **Albertus Beishuizen:** Writing – review & editing, Investigation. **Wytze J. Vermeijden:** Writing – review & editing, Investigation. **Francois H.M. Kornips:** Writing – review & editing, Investigation. **Norbert A. Foudraine:** Writing – review & editing, Conceptualization. **Jeannette Hofmeijer:** Writing – review & editing, Supervision, Project administration, Conceptualization. **Michel J.A.M. van Putten:** Writing – review & editing, Writing – original draft, Supervision, Data curation, Conceptualization.

## Declaration of competing interest

The authors declare the following financial interests/personal relationships which may be considered as potential competing interests: MVP is co-founder of Clinical Science Systems, a manufacturer of clinical EEG software. The remaining authors have no conflicts of interest.
